# Progress in molecular diagnosis and treatment of chronic mucocutaneous candidiasis

**DOI:** 10.3389/fimmu.2024.1343138

**Published:** 2024-01-24

**Authors:** Danrui Jing, Guanzhao Liang, Xiaofang Li, Weida Liu

**Affiliations:** ^1^ Department of Medical Mycology, Hospital for Skin Diseases, Institute of Dermatology, Chinese Academy of Medical Sciences & Peking Union Medical College, Nanjing, China; ^2^ Jiangsu Key Laboratory of Molecular Biology for Skin Diseases and STIs, Nanjing, China; ^3^ Chinese Academy of Medical Sciences Collection Center of Pathogen Microorganisms-D (CAMS-CCPM-D), Nanjing, China; ^4^ Center for Global Health, School of Public Health, Nanjing Medical University, Nanjing, China

**Keywords:** chronic mucocutaneous candidiasis, progress, molecular diagnostics, therapy, prognosis

## Abstract

Chronic mucocutaneous candidiasis (CMC) is characterized by recurrent or persistent infections with *Candida* of the skin, nails, and mucous membrane. It is a rare and severe disease resulting from autoimmune defects or immune dysregulations. Nonetheless, the diagnosis and treatment of CMC still pose significant challenges. Erroneous or delayed diagnoses remain prevalent, while the long-term utility of traditional antifungals often elicits adverse reactions and promotes the development of acquired resistance. Furthermore, disease relapse can occur during treatment with traditional antifungals. In this review, we delineate the advancements in molecular diagnostic and therapeutic approaches to CMC. Genetic and biomolecular analyses are increasingly employed as adjuncts to clinical manifestations and fungal examinations for accurate diagnosis. Simultaneously, a range of therapeutic interventions, including Janus kinase (JAK) inhibitors, hematopoietic stem cell transplantation (HSCT), cytokines therapy, novel antifungal agents, and histone deacetylase (HDAC) inhibitors, have been integrated into clinical practice. We aim to explore insights into early confirmation of CMC as well as novel therapeutic options for these patients.

## 1 Introduction

Chronic mucocutaneous candidiasis (CMC) comprises a heterogeneous group of syndromes characterized by recurrent or persistent infections of the skin, nails, and mucous membrane caused by *Candida* species, mainly *C. albicans*. It is a primary immunodeficiency disorder arising from autoimmune defects or immune dysregulations, with numerous confirmed or potentially relevant genetic and biomolecular factors associated with this condition. Notably, gain-of-function mutations (GOF) in the signal transducer and activator of transcription 1 (*STAT1*) gene have been identified as the underlying cause in approximately half of CMC patients (1). CMC is also among the most frequent infections in patients with autoimmune polyendocrinopathy candidiasis ectodermal dysplasia (APECED) syndrome and hyper-IgE syndrome (HIES). This disease is rare and severe, not only significantly affecting patients’ quality of life, but also resulting in a substantial reduction of life expectancy due to potential development in combination with severe combined immunodeficiency (SCID) or malignances (1, 2). However, the diagnosis of CMC can be challenging due to atypical or polymorphic clinical manifestations, which may lead to delays in confirming this disorder (3). The recent molecular findings have greatly enhanced our understanding of the pathogenic mechanisms underlying CMC, compensating for limitations associated with clinical manifestations and fungal examination, thereby contributing to substantial advancements in early diagnosis.

At present, the majority of CMC patients necessitate long-term antifungal therapy, predominantly systemic. The first-line agents are azole antifungal drugs, with fluconazole serving as the prototypical example, followed by itraconazole and posaconazole (1). Second- or third-line treatments, including voriconazole, echinocandins, terbinafine, or liposomal amphotericin B, are occasionally required (4). However, prolonged exposure to antifungals can facilitate the development of resistant *Candida* strains, and the high prevalence of antifungal resistance in CMC remains a significant challenge (4–6). In a large-scale cohort study, approximately 39% (78/202) of patients undergoing long-term antifungal therapy demonstrated clinical resistance to at least one antifungal agent, particularly azoles (4). In addition, patients with resistance to antifungals showed more severe phenotypes including recurrent pneumonia, systemic fungal infections and increased mortality (4). Thus, prompt and effective intervention is crucial in improving patients’ quality of life, and novel treatment strategies are warranted to enhance the prognosis. In this study, we conducted a comprehensive literature review to delineate the advancements in the molecular diagnosis and treatment of CMC, aiming to furnish a wider array of options for researchers and clinicians.

## 2 Molecular diagnosis

### 2.1 Genetic tests

Over the past decade, a growing number of immunodeficiencies have been identified that cause CMC, many of which are associated with impaired development of Th17 and Treg defects, rendering individuals susceptible to *Candida* infections (7). Mutations in genes including IL17F, IL17RA, IL17RC, ACT1 (TRAF3IP2), and RORC have been identified in some CMC patients (7–9). Additionally, mutations affecting proteins involved in the signaling pathway of IL-17 including *STAT1*, *CARD9*, *STAT3*, *TYK2*, and *DOCK8* have also been observed (7). Among them, *STAT1* GOF mutations represent the most prevalent genetic cause of inherited CMC as well as various infectious and autoimmune diseases along with cerebral aneurysms and carcinomas with a poor outcome (4). Toubiana et al. identified 76 mutations in this gene associated with CMC and other researchers also observed additional novel mutations (4, 10–12). Furthermore, APECED syndrome with biallelic mutations in the *AIRE* gene and HIES with dominant-negative mutations in the *STAT3* gene are also characterized by increased susceptibility to fungal infections, which may manifest as CMC (13–16). The number or function of Tregs was observed to be diminished in patients with CMC those carrying *STAT1* GOF mutations (17). Therefore, when clinical suspicion arises for CMC, the aforementioned genes can be examined to aid diagnosis.

Xie et al. reported a patient initially misdiagnosed as fissured tongue and oral candidiasis, which was later confirmed as CMC after 18 years, via whole exome sequencing (WES) analysis revealing an IL-17RC mutation (3). Additionally, in cohort analysis, targeted panel sequencing played an important role in being a cost-effective first-line genetic screening method that facilitates the identification of mutations even in patients with atypical clinical presentations. In this way, more than one-third of patients diagnosed with CMC were found to harbor genetic defects, including 23 *STAT1* and 4 *CARD9* mutations (13). Thus, the significance of genetic tests is underscored in these reports, as they facilitate precise timing of treatment and enable early prediction and detection of associated complications in the management of CMC (18).

### 2.2 Anti-IL-17A/F(IL-22) antibodies detecting

High titer-neutralizing anticytokine antibodies (ACAAs) have been identified in patients with severe or refractory fungal infections in recent years (19). IL-17A and F play crucial roles in the adaptive immune system’s response to fungal infections, which are generated by CD4 (specifically Th17) cells and stimulate epithelial cells to express antimicrobial peptides, as well as increase neutrophil trafficking (19). Consequently, autoantibodies to IL-17A and IL-17F might be associated with the CMC phenotype. A study involving 33 patients with autoimmune polyendocrine syndrome type 1 (APS-1, also known as APECED) presenting with CMC detected high titers of ACAAs against IL-17A, IL-17F, and/or IL-22, and no such autoantibodies were observed in 37 healthy controls or the 103 patients with other autoimmune disorders (20). Another multicenter survey revealed that a significant proportion (41%) of over 150 APECED patients with CMC exhibited autoantibodies to IL-17A, while higher percentages were observed for IL-17F (75%) and/or IL-22 (91%) (21). These findings were particularly prominent in APECED patients with CMC whereas normal responses and infrequent presence of autoantibodies were noted in APECED patients without CMC (21).

Screening of ACAAs thus will pave the way for the diagnosis of CMC, particularly in patients without established autoimmune etiologies. The failure to recognize ACAAs as an underlying cause of secondary immunodeficiencies may contribute to the challenges in identifying these patients. ELISA and multiplex particle-based flow cytometry, which have been employed to detect antibodies capable of neutralizing IL-17 and IL-22, with confirmation of antibody specificity by Western blot, could be advantageous (20).

### 2.3 Cytokine profiling in peripheral blood and RNA-seq analysis

Prolonged excess production of some cytokines may potentially increase the vulnerability to mucosal fungal infections in specific environments. Break and colleagues (22) recently uncovered that an increase in the production of interferon-γ (IFN-γ) by mucosal T cells promotes IFN-γ–dependent epithelial barrier disruption and mucosal fungal susceptibility, ultimately leading to candidiasis in APECED. Wang et al. (10) performed cytokine profiling in CMC patients with specific *STAT1* GOF mutation and discovered elevated levels of serum IFN-α compared with the wild-type group. This finding is consistent with previous studies demonstrating that CMC patients with *STAT1* GOF mutations exhibit impaired dephosphorylation of STAT1, leading to hyperphosphorylation of STAT1 and persistent response to IFNs and IL-27, which act as inhibitors for Th17 cells (23, 24).

In addition, the multidrug-resistant pathogen *Candida auris*, being one of only four pathogens in the critical group in the WHO fungal priority pathogens list, was identified to be the causative agent of CMC in a father and daughter with TP63-associated ectodermal dysplasia (ED) (25). In this study, RNA-Seq analysis was utilized to detect this unique non-albicans *Candida* species (25), which could also be considered a valuable tool for identifying rare and frequently misidentified pathogens in CMC.

## 3 Treatment

### 3.1 Hematopoietic stem cell transplantation (HSCT)

HSCT is currently the only curative approach for severe or refractory CMC, particularly in patients with STAT1 GOF mutation. Nonetheless, associated risks like infection, graft-versus-host disease (GVHD), and mortality following secondary transplantation continue to pose significant challenges (1). A systemic review described 25 patients with STAT1 GOF mutation receiving HSCT, among which eleven died several months later, seven experienced resolution of disease-related symptoms and four demonstrated immune reconstitutions (26). Prompt recognition and diagnosis of STAT1 GOF mutation are vital, as patients who undergo early transplantation are more likely to achieve success in preventing severe complications and enhancing overall survival (27). Borgström et al. reported a patient with *STAT1* GOF mutation who underwent HSCT following unsuccessful treatment with JAK inhibitors, and his clinical status has improved considerably, showing no evidence of *C. albicans* infection, GVHD, or gastrointestinal symptoms even after one year (28). Kiykim et al. also reported a successful HSCT treatment in a three-year-old girl with *STAT1* GOF who had a rescue of Th17 differentiation (29).

IFN-γ played an important role in inducing high rates of graft failure. Emapalumab, a human IFN-γ–blocking monoclonal antibody, has been employed as a pretransplant immunosuppressive strategy in a patient with a *STAT1* GOF mutation to facilitate successful engraftment by shielding the graft from inflammation during the process (30). Furthermore, patients with *CARD9* deficiency also achieved complete clinical remission underlying invasive fungal infections after HSCT (31).

### 3.2 JAK inhibitors

In recent years, Janus kinase (JAK) inhibitors have been used as a favorably responded therapy in several cases of CMC. By targeting the JAK/STAT axis, JAK inhibitors exhibit promise as drug candidates, particularly for patients with GOF mutations in *STAT1* or *STAT3*. Ruxolitinib, a JAK1/2 inhibitor, has shown efficacy in inhibiting the hyper-phosphorylation and hyper-responsiveness to interferon of STAT1, normalizing Th1 and follicular T helper cell responses, and improving Th17 differentiation, ultimately leading to the cure of a child with autoimmune cytopenias and CMC (32). This small molecule also plays a role in rescuing terminal maturation of NK cells and partially restoring perforin expression in NK cells, thereby salvaging NK cell cytotoxic function (33). Olivier et al (34) reported a case of partial clinical remission in a patient treated with ruxolitinib. Upon reviewing 20 cases of *STAT1* GOF mutation treated with JAK inhibitors, they found that the majority (16/17) presenting a clinical picture of CMC were treated with ruxolitinib, among which eleven showed improvement or a resolution of this disorder. The effectiveness of improving the clinical status of ruxolitinib has been demonstrated in other clinical studies (n = 33 cases), mainly in children with *STAT1* GOF mutation (28).

However, other reports suggest that the partial improvement of immune dysregulation and the remaining susceptibility to opportunistic infection after ruxolitinib treatment may indicate the role to merely serve as a bridge before hematopoietic stem cell transplantation (HSCT) of ruxolitinib (35). Ruxolitinib could improve disease management and immune dysregulation profile to reduce the risk of adverse outcomes in HSCT (36). Furthermore, the dysregulation of STAT1 phosphorylation kinetics, gene expression related to multiple immune pathways, and TH17 deficiency were found to be improved or persisted after ruxolitinib treatment but completely normalized following HSCT (36).

Baricitinib, another selective JAK1/2 inhibitor, demonstrates an action mechanism resembling ruxolitinib, albeit with reduced potency, and displays slightly enhanced selectivity for JAK1 and JAK2 (28). Upon the administration of baricitinib, researchers utilized mass cytometry to examine cell surface markers, which reflected an augmented functional phenotype and a diminished inflammatory response. The patient treated with baricitinib exhibited a notable clinical improvement, and the immune response to *C. albicans* was exacerbated after seven weeks of treatment (28). Additional patient with a *STAT1* GOF mutation also showed significant improvement with no adverse events following administration of baricitinib, and the capacity to produce IL17A, IL-17F, and IL-22 of PBMCs was enhanced (37).

Overall, after JAK inhibitors, the severe clinical manifestations of most patients can be significantly improved, and autoimmune or immunodysregulation status can be partially restored. Transaminase levels and complete blood count should be monitored frequently to adjust the dose of JAK inhibitors in case abnormalities occur and acyclovir prophylaxis may be prescribed due to the risk of herpes virus infections (38). The most effective dose and bioavailability are still needed to be investigated (38).

### 3.3 Cytokine therapy

Cytokines can promote host defense and may therefore be potentially valuable for immunomodulation during infections. GM-CSF and G-CSF treatments, which have been proposed as a way of enhancing IL-17 T-cell differentiation (39–41), could serve as adjunctive therapy in combination with antifungal therapy, resulting in improved clinical and mycological outcomes without adverse events in patients with refractory mucosal candidiasis (42). GM-CSF produced by PBMC is proposed to be diminished in *CARD9* deficient patients (43). The utility of GM-CSF as adjunctive therapy was observed to achieve complete clinical remission in a patient with *CARD9* mutation presenting with CNS candidiasis (44). In 1995, Shahar et al. first reported that a CMC patient resistant to conventional antifungals experienced significant clinical benefits after receiving GM-CSF treatment (41). However, despite these promising findings, only one of five patients receiving G-CSF demonstrated a marked improvement in a study (4).

### 3.4 Novel antifungal agents or dosage forms

Rezafungin is a novel echinocandin specifically designed for the treatment of invasive candidemia and invasive *Candida* infections of hospitalized patients (6). Melenotte et al. (6) reported a favorable clinical outcome in a patient with *STAT1* GOF CMC who was resistant to azoles, successfully treated with rezafungin without any clinical or biological adverse effects. The advantage of its once-weekly administration not only enhances the quality of life for patients but also provides novel perspectives for antifungal prophylaxis.

The Cochleated AMB, a lipid-nanocrystal formulation utilized for targeted oral delivery of AMB, demonstrated efficacy in patients with azole-resistant CMC and showed exceptional tolerability, safety, and systemic absorption (5). Tetrazole VT-1598 represents another novel, broad-spectrum, and highly selective fungal CYP51 inhibitor (45). *In vivo* studies corroborated the potent inhibitory effect of VT-1598 on fluconazole-susceptible and -resistant *C. albicans* in IL-17 signaling-deficient mice, even following a prolonged washout period or at low doses. *In vitro* assessments disclosed the elimination of VT-1598 on mucosal *Candida* strains including fluconazole-resistant strains (45). These reports provide potential drug candidates that are promising and can significantly improve treatment options for CMC.

### 3.5 Histone deacetylase (HDAC) inhibitors

The potential of HDAC inhibitors to provide novel targets for CMC therapies has been demonstrated in *in vitro* studies. The inhibition of HDACs in cells with *STAT1* GOF mutations has been shown to restore normal *STAT3*-dependent gene expression, which triggers the production of Th-17 cytokines that mediate protective immunity in CMC (24). Pan-HDAC inhibitors have a profound influence in inhibiting innate and adaptive cytokines, and specific HDAC inhibitors, such as entinostat and RGFP966, appear to be the optimal therapeutic candidates due to their capacity to restore IL-22 production and decrease *STAT1* phosphorylation (46). However, the function of HDAC inhibitors whether induce a possible risk of secondary infections needs to be investigated.

## 4 Conclusions

CMC is a rare and severe primary immunodeficiency characterized by a broad clinical phenotype with a poor prognosis. Early diagnosis is of utmost importance since it not only benefits the patients by providing prompt treatment but also enhances overall survival rates. The diverse immune dysregulations emphasize the significance of implementing genetic or biomolecular tests. Novel treatment strategies, which are based on rectifying the underlying mutations and affected pathways, such as reducing hyperphosphorylation of *STAT1* and restoring Th17 function, are being developed. Further innovative approaches are needed to be investigated.

## Author contributions

DJ: Writing – original draft, Writing – review & editing. GL: Supervision, Writing – review & editing. XL: Supervision, Writing – review & editing, Conceptualization, Funding acquisition. WL: Project administration, Supervision, Writing – review & editing.
